# Bayesian multiple membership multiple classification logistic regression model on student performance with random effects in university instructors and majors

**DOI:** 10.1371/journal.pone.0227343

**Published:** 2020-01-30

**Authors:** Elsa Vazquez Arreola, Jeffrey R. Wilson

**Affiliations:** 1 School of Mathematical and Statistical Sciences, Arizona State University, Tempe, AZ, United States of America; 2 Department of Economics, Arizona State University, Tempe, AZ, United States of America; Northwestern University, UNITED STATES

## Abstract

Educational success measured by retention leading to graduation is an essential component of any academic institution. As such, identifying the factors that contribute significantly to success and addressing those factors that result in poor performances are important exercises. By success, we mean obtaining a semester GPA of 3.0 or better and a GPA of 2.0 or better. We identified these factors and related challenges through analytical models based on student performance. A large dataset obtained from a large state university over three consecutive semesters was utilized. At each semester, GPAs were nested within students and students were taking classes from multiple instructors and pursuing a specific major. Thus, we used multiple membership multiple classification (MMMC) Bayesian logistic regression models with random effects for instructors and majors to model success. The complexity of the analysis due to multiple membership modeling and a large number of random effects necessitated the use of Bayesian analysis. These Bayesian models identified factors affecting academic performance of college students while accounting for university instructors and majors as random effects. In particular, the models adjust for residency status, academic level, number of classes, student athletes, and disability residence services. Instructors and majors accounted for a significant proportion of students’ academic success, and served as key indicators of retention and graduation rates. They are embedded within the processes of university recruitment and competition for the best students.

## Introduction

At institutions of higher education, undergraduate student academic performance is a major concern for the administration. Monitoring academic progress made in student retention and graduation rates is key to funding, expansion of programs, and improving performance. However, although this aspect of undergraduate student services has seen increased budgetary attention, there are still unexplained aspects within these programs. Several of these monitoring programs have benefitted from the aid of predictive modeling and the use of random effects to account for the unmeasurable effects [1].

Several researchers have investigated what factors drive college students’ academic performance and what factors impact persistence to graduation. Most studies define academic performance based on course grades or cumulative GPA. Stewart, Lim and Kim [2] found that there was a strong correlation between first semester college GPA and college persistence. Allen and Robbins [3] concluded that in 4-year colleges, students’ first year academic performances had a large effect on timely degree completion.

Fischer, Hilton, Robinson, et al. [4] conducted a study to assess whether the adoption of no-cost open digital textbooks had an impact on students’ completion of courses, class achievement, and enrollment intensity. They used data collected from 15 courses at four 4-year colleges and six community colleges. One of their outcomes measured whether students passed their courses with a C or better. They used chi-square tests (bivariate analysis) to obtain the association between passing courses with a C or better and using open digital textbooks. They analyzed each course separately while acknowledging that there is extra variation present when comparing success across classes. Such variation is also present in the level of class difficulty across faculty and within departments.

Lepp, Barkley and Karpinsky [5] studied self-reported data obtained from 526 undergraduate students across 82 majors. They examined the relationship between cell-phone use and academic performance while controlling for high school GPA, self-efficacy for self-regulated learning, self-efficacy for academic achievement, gender, cigarette use, class standing, and major. They obtained a predictive model of academic performance measured by college GPA using these covariates with 82 majors as fixed effects. The 82 majors were grouped, as otherwise there would be 81 additional parameters in the model.

Faculty is an important part of the educational process. Deutsch [6] examined how part-time faculty impacts retention and graduation rates using the Integrated Post-Secondary Education Data System (IPEDS). Deutsch fitted a longitudinal model with fixed effects and found that an institution’s proportion of part-time faculty is not statistically significant when one studies retention and graduation rates with other moderators.

Hutto [7] studied course retention (the completion of a course with a grade of C or higher) and found that it had an impact on degree completion. Further, he reported to have found a significant relation between course retention and faculty employment status. Bettinger and Long [8] found that adjunct faculty and graduate teaching assistants impact the likelihood of enrollment and success in different ways. Using value-added models, they found that taking a course from an adjunct professor or graduate student had a negative impact on a student’s future performance. Value-added models have been used by the American Statistical Association (ASA) for educational assessment [9]. Ran and Xu [10] contrasted the effects of tenured professors, tenure-track professors, long-term adjunct professors, and short-term adjunct professors on student academic outcomes. They found that adjunct professors have a positive impact on grades for introductory courses but a negative impact on grades in subsequent courses.

Fischer, Hilton, and Robinson [4] found that course difficulty naturally varies by instructor, by student major, and these have varying effects on students’ academic outcomes. Some researchers modeled these differences by analyzing academic outcomes for each course or major separately. Others have classified instructors as a fixed effect, separating them into different groups based on certain characteristics. However, fixed effects models limit researchers from extrapolating beyond the scope of the data. On the other hand, the use of random effects models allows one to extrapolate. In particular, when these fixed factors consist of several categories, it is common to make use of random effects instead of fixed effects. In this study, we want to investigate how the multiple instructors that students take classes with and the major the students pursue during a particular semester affect their academic performance, measured by their semester’s GPAs, by treating instructors and majors as random effects. Instructors and majors are both clustering variables or classifications for students. The variance of these random effects (majors and instructors) allows one to measure the variability in academic success that can be attributed to instructors and to majors.

We analyze three semesters of data that have a non-hierarchical multilevel data structure, where semester’s GPAs are completely nested within students while students are contained into a cross-classification of instructors and majors. Students are said to be cross-classified by instructors and majors since they are clustered within both classifications, but instructors are not purely nested within majors and majors are not purely clustered within instructors. This combination of random effects—with students usually belonging to two or more different instructors and one particular major at each semester—gives rise to Multiple Membership Multiple Classification (MMMC) models also known as Cross-Classified Multiple Membership (CCMM) models [11,12]. The use of MMMC logistic regression model accounts for the varying impact of instructors and majors on success. Fitting separate models ignores the correlation between instructors and majors and assumes that the performance of students is the same across instructors and across majors; certainly, this is not the case [13].

In this paper, we fit MMMC logistic regression models to decipher academic success. Academic success is measured on a binary scale based on one’s semester grade of B or better in one set of models and then one semester’s grade of C or better in another set of models. We choose a B or better as there is a multitude of opportunities for undergraduates if they complete with a B or better. Opportunities include admission to graduate school, medical school, law school, or other graduate programs, as well as eligibility for scholarships and research grants towards present or future studies. One may equally argue that a C or better is an important point to dichotomize, as it is the threshold for academic probation and has been used as a retention measure which impacts graduation [7]. Obviously, successfully completing all courses with a C or higher would increase the likelihood of degree completion [7]. We see merit in both cut points and as such, fit models to both target variables.

It is very likely and conceivable that the interactions between students and instructors or the delivery mode of the lectures by instructors, or the comradery among students in the classes, or the structures of the major may have an impact on any given student’s performance during a semester. More importantly, the student’s semester GPA performance is made up of interrelated factors, some having a direct impact and some having an indirect impact. Some students have different instructors, and some students may change majors. In addition, instructors teach in different majors. Such cases result in multiple memberships of students within instructors, as they are not fully nested since students don’t receive all classes from the same instructors and are cross-classified by major. This cross-classification arises because different sets of instructors don't all teach courses corresponding to the same single major [11,12]. As such, we use MMMC logistic regression models to fit to these data. In addition, we make use of prior information available about students to model success.

This complexity of the non-hierarchical multilevel structure of the data necessitates the use of Bayesian parameter estimates of the regression coefficients from the posterior distribution. The posterior is a combination of the binomial likelihood function (as it is a binary outcome) of the data and the prior distributions of the parameters. We use normal priors for regression coefficients based on information we obtained from other studies. We use inverse gamma priors for random effects’ variances as such priors let us draw parameter values that are always positive. Moreover, the complexity of the model results in the frequentist approach not converging at times (as is the cases with these data). The MMMC logistic regression model with Bayesian parameter estimates identifies the key factors of academic success, while accounting for the unmeasurable effects due to instructors and majors. We were not able to determine if at any given semester, students were also multiple members of majors from the dataset, so for students who double majored, our results did not account for their situation and only consider single majors.

Section 2 provides a review of the hierarchical logistic regression model. In Section 3, we present the MMMC logistic regression model with Bayes estimates, which addresses the effects of instructors and majors. In Section 4, an analysis of three consecutive semesters of data from a large state university is explored. Some conclusions and discussions are given in Section 5.

## Background

In the analysis of binary data, there is a straightforward approach to model independent observations as opposed to modeling correlated observations. When the outcomes are obtained on account of an independent mechanism, one usually fits the standard logistic regression model with *K* covariates such that

$$logit(p)=ln\left(\frac{p}{1-p}\right)=\beta_{0}+\beta_{1}x_{1}+\beta_{2}x_{2}+\cdots +\beta_{K}x_{K}$$ (1)

where *p* denotes the probability of success, *β*_*i*_ is the regression coefficient for the covariate *x*_*i*_ and *i* = 1,2,…,*K*. It is customary to obtain estimates of the regression coefficients ${\hat{\beta }}_{i}$ based on the method of maximum likelihood.

However, in the analysis of nested or hierarchical data the independence assumption is no longer applicable so the maximum likelihood is not attainable. In addition, the hierarchical structure brings a measure of the intraclass correlation at each level of the hierarchy. Such is certainly the case with the three consecutive semesters of university performance data. In that case, GPAs are nested within students, students are nested within majors, and are multiple members of instructors. As such, the standard logistic regression model is not appropriate. It ignores the intraclass correlation inherent due to the multilevel structure. The data structure demands a model that incorporates the correlation due to the hierarchical structure of the data and the multiple memberships. Such multilevel structure is common in many fields of research, especially education. However, a standard logistic regression model ignores the clustering, and as such is likely to lead to inferences made that are not valid [14–16]. Thus, an adequate multilevel model must account for the correlation inherent at the different levels of the hierarchy [1]. This paper presents a fit of MMMC logistic regression models. Such models allow one to account for multiple sources of variation due to the multiple levels of the hierarchy that may impact the response but are not directly measured [17].

We fit these models through the use of three consecutive semesters of performance data. The semester serves as a proxy for time but is treated as a fixed effect. It allows us to address the sustained performance of students in a variety of courses. The data structure is not completely nested; it consists of GPAs nested within students and students completely nested within majors and a multiple membership within instructors. Single membership for instructors would require that a student took all of his/her classes from the same instructor. Then, the student’s semester GPA would be affected by a single instructor, as the case in elementary schools or home schooling.

Consider a student’s GPA, $y_{i|j}^{*}$, measured on a continuous scale. We assume that $y_{i|j}^{*}$ has an error term, $\varepsilon_{i|j}^{*}$, which follows a logistic distribution with mean zero and variance $\frac{\pi^{2}}{3}$. We dichotomize $y_{i|j}^{*}$ to obtain a binary variable *y*_*i*|*j*_ where *y*_*i*|*j*_ has value one when $y_{i|j}^{*}\geq 3$ and 0 otherwise. Then, a single membership logistic regression model with instructors as random effects is,

$$logit\left[P(y_{i|j}^{*}\geq 3)\right]=\beta_{0}+\beta_{1}X_{i1}+\beta_{2}X_{i2}+\cdots +\beta_{k}X_{iK}+\gamma_{j}$$ (2)

where (2) denotes the log of the odds that student *i* succeeds in obtaining a GPA equal to or greater than 3.0 with instructor *j*. The regression coefficient *β*_0_ represents the intercept, while *β*_1_,*β*_2_,…,*β*_*K*_ are the coefficients of the student-level covariates *X*_*i*1_,*X*_*i*2_,…,*X*_*iK*_. The random effect for instructor *j* is given by *γ*_*j*_ and is distributed as $N(0,\sigma_{\gamma }^{2})$ with $\sigma_{\gamma }^{2}$ representing the variance among the instructors. Therefore, the probability of success for student *i*, taking classes with instructor *j*, is

$$P(y_{i|j}^{*}\geq 3)=\frac{e^{\beta_{0}+\beta_{1}X_{i1}+\beta_{2}X_{i2}+\cdots +\beta_{K}X_{iK}+\gamma_{j}}}{1+e^{\beta_{0}+\beta_{1}X_{i1}+\beta_{2}X_{i2}+\cdots +\beta_{K}X_{iK}+\gamma_{j}}}.$$

The total response variance for such two-level structure for $y_{i|j}^{*}$ is $var(y_{i|j}^{*})=\sigma_{\gamma }^{2}+\frac{\pi^{2}}{3}$.

Browne, Subramanian, Jones and Goldstein [13] suggested a variance partition coefficient (VPC) for such latent logistic regression models to report the proportion of the response variance unexplained by covariates in the model that can be attributed to each level in the hierarchy. Thus, the VPC for instructors is ${VPC}_{instructors}=\frac{\sigma_{\gamma }^{2}}{\sigma_{\gamma }^{2}+\frac{\pi^{2}}{3}}$ where $\sigma_{\gamma }^{2}$ is the variance at instructor-level and $\frac{\pi^{2}}{3}$ is the variance at the observational level, under the assumption that $\varepsilon_{i|j}^{*}∼logistic\left(0,\frac{\pi^{2}}{3}\right)$ [18].

However, at the university level of education, it is inconceivable that a student will take all courses from one instructor at any given semester. This necessitates a multiple membership model to account for the nesting of students within more than one instructor. Ignoring the fact that, at each semester, students take classes with more than one instructor and fitting a model that accounts for random effects of only one instructor per semester would lead to inaccurate parameter estimates of the between-instructors variance in academic success [19]. It would mean that students’ semester’s GPA would be modeled as only having been affected by that one instructor considered in the model and the potential effects of the multiple instructors would be ignored [20]. Also, it is necessary to account for the effect of majors in students’ academic performance, because of this, it is essential to fit multiple membership multiple classification models to our data. If the sources of variation due to majors are not included or if we conduct separate instructors’ models, the standard errors of the regression parameters’ estimators are underestimated, thereby leading to incorrect conclusions about statistical significance of the fixed effects [19, 21]. When one level of the cross-classification is ignored, either instructors or majors, the variance of the random effects for the non-ignored classification is generally overestimated [22]. If separate models, one for instructors and another for majors are fit, the variance components obtained from these separate models are not reliable [23].

In this paper, we fit multilevel logistic regression models with multiple memberships and cross-classifications for the instructors’ and majors’ levels. The additional parameters in such models give rise to an increased complexity in the fit of the model and at such times the frequentist model does not necessarily converge. This requires the fit of a Bayesian model to study academic success with multiple memberships while making use of the random effects due to instructors and majors.

## A Bayesian multiple membership multiple classification model for success

### Bayesian model

**Fig 1:**
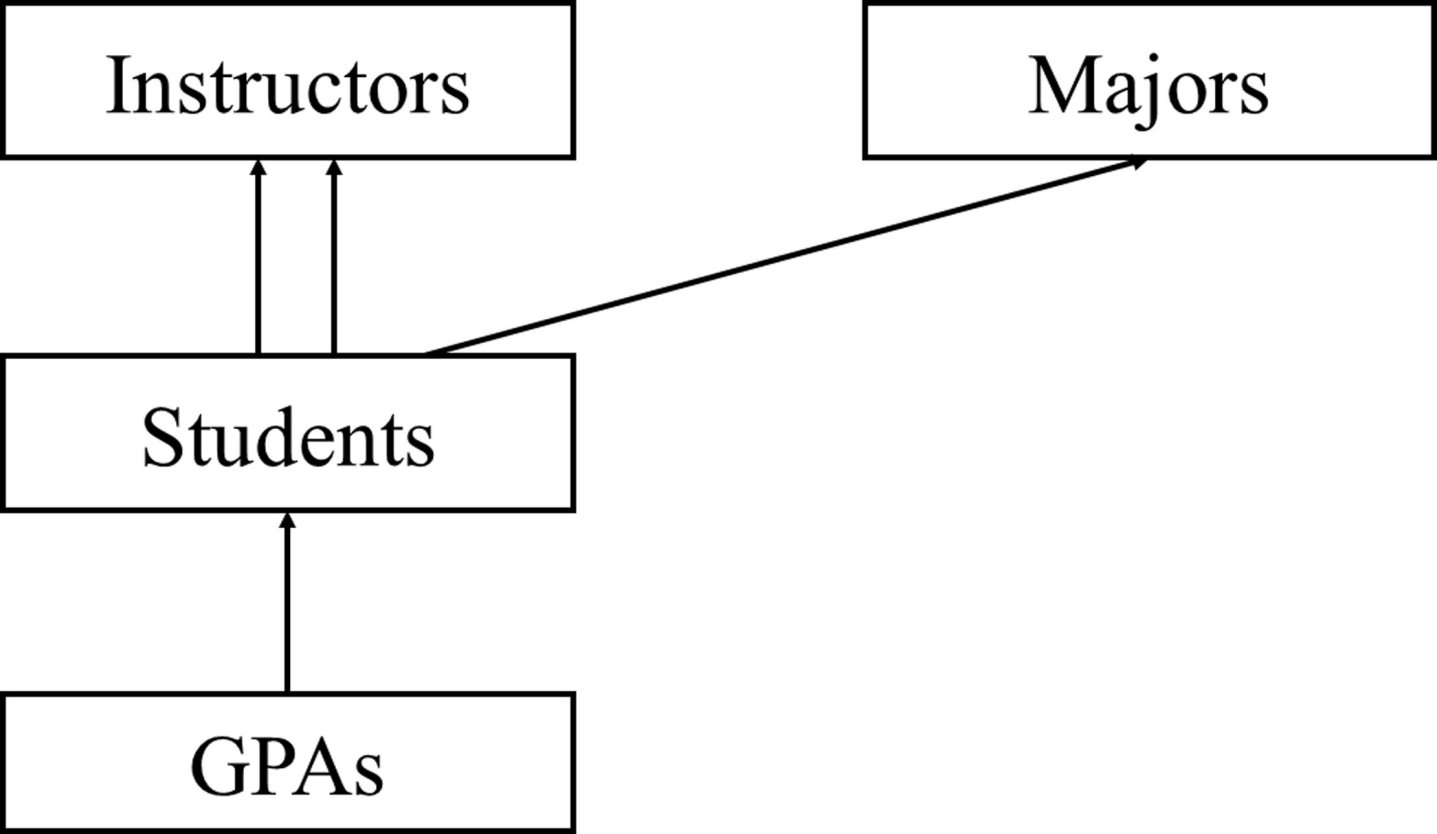
Multiple membership multiple classification GPAs, students, majors, and instructors.

Though multiple membership multiple classification (cross-classified multiple membership) is a common multilevel data structure, it is often ignored in the model analysis due to the added complexity it brings to the computations. In our study data, which consisted of three different semesters, students took courses from more than one instructor and were pursuing different majors. Thus, we have many levels of classifications and memberships. At level-1, we have semester GPAs (our outcome of interest), which are completely nested within students at level-2, while students are completely nested within majors (classification 3) and are multiple-members of instructors (classification 4). The instructors and the majors also have unmeasurable effects on the overall performance of the student. However, it is common, when faced with such phenomenon, for researchers to use models where instructors and majors are treated as fixed effects or fit separate models for each course or each major. But, such approaches negate extrapolation beyond the scope of the data. The multilevel non-hierarchical structure of the data gives rise to a multiple membership multiple classification model (MMMC), also known as cross-classified multiple membership model. Thus, we present an alternative model. A model that accounts for all effects simultaneously while incorporating the cross-classified multiple membership structure of the data. Our dataset consisted of three semesters of data, for some students only one semester of data is available, while for others we had data for two or three semesters. Fig 1 provides a schematic diagram of the overall structure of the model [11]. It shows that GPAs are fully nested within students and students are nested fully within majors as indicated by the single solid arrows, but not completely nested within instructors represented by the double solid arrows.

**Fig 2:**
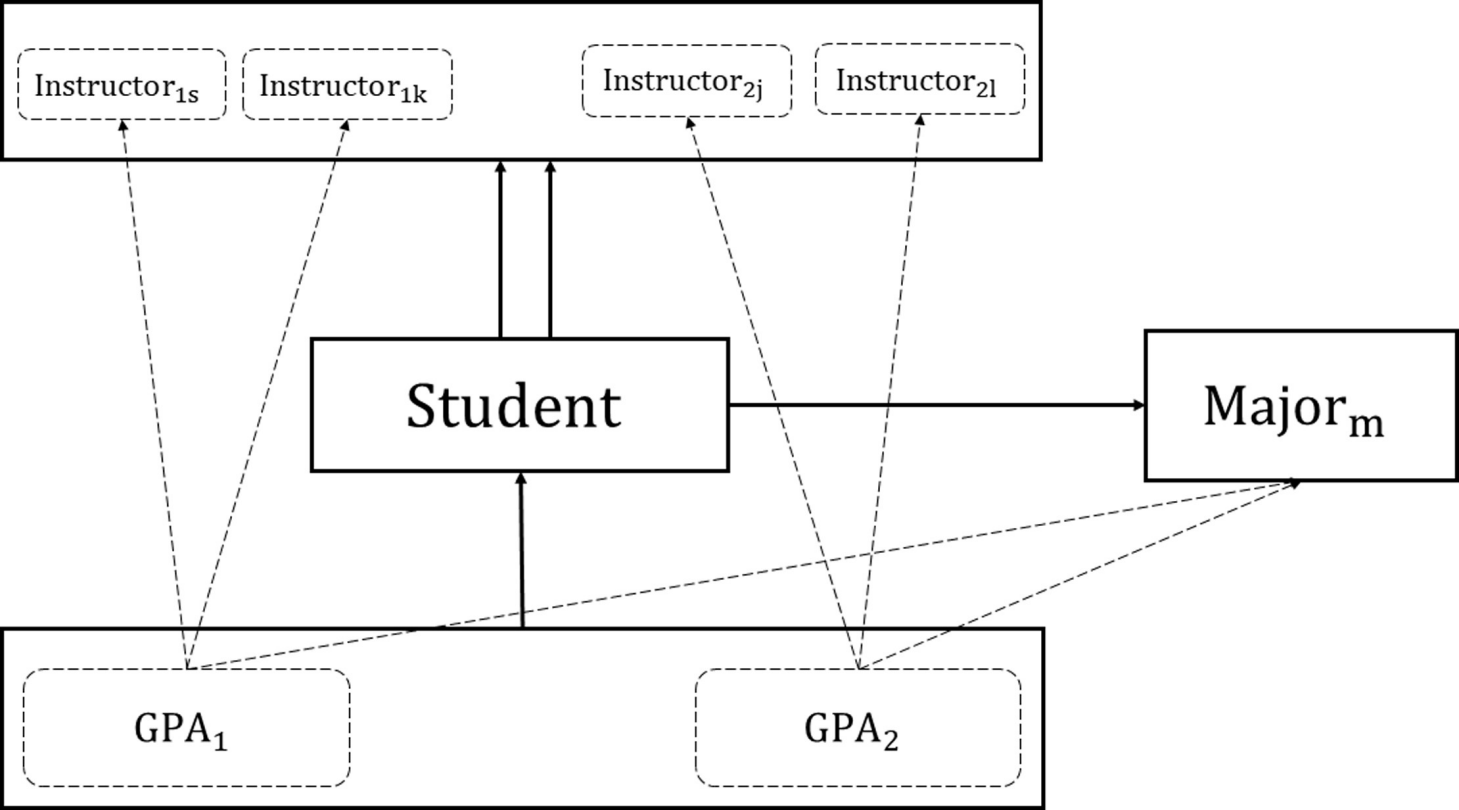
Data structure for students with two semesters of data, two instructors at each semester and same major in both semesters.

As explained earlier, there are students who only have one semester of data and there are others who have two or three. We provide a more detailed illustration of the multilevel structure for students with data in the first two semesters in Fig 2. We follow Choi and Wilson’s [24] graphical representation and use an example where students have two instructors at each semester and the same major both semesters. The solid rectangles and arrows represent the multiple levels and classifications in our data: GPAs, students, instructors and majors. GPAs are completely nested within students (single solid arrow between GPA and student levels), students are multiple members of instructors (two solid arrows between student and instructors’ level) and are completely nested within majors (single solid arrow between student and major levels). Dotted rectangles within the GPA level indicate GPAs at specific semesters. Within the instructor level, particular instructors are also represented by dotted rectangles. Furthermore, dotted arrows from *GPA*_1_ to *Instructor*_1*s*_ and *Instructor*_1*k*_ indicate the influence of these two instructors, *s* and *k*, on students’ GPA at semester 1, and dotted arrows from *GPA*_2_ to *Instructor*_2*j*_ and *Instructor*_2*l*_ show that GPA at semester 2 is affected by instructors *j* and *l*. Similarly, dotted arrows from *GPA*_1_ and *GPA*_2_ to *major*_*m*_ indicate the effect of major *m* on both GPAs. Fig 2 can be modified to illustrate the structure for when students only have one instructor or more than two instructors, and when they have data collected only at one semester or at three. It can also be altered to show whether students changed majors from one semester to another. However, when fitting our models for any given semester, we only accounted for the effects of instructors that the student took classes with and the major that the student was pursuing at that specific semester. Our model did not consider effects of instructors with whom the student took classes in previous or future semesters, nor did it account for impacts of majors that the student pursued before or after that semester.

To address the research in this paper, we assumed that at each semester we had GPAs nested within students, students cross-classified by instructors and majors, and that students were multiple members of instructors and completely nested within majors. Thus, we fitted a multiple membership multiple classification logistic regression model that included random effects for students to account for the longitudinal aspect of the data when students had data for two or three semesters [11,22,24]

$$logit\;P(y_{i_{t}|i,Ins(i_{t}),maj(m_{t})}^{*}\geq 3)=\beta_{0}+\beta_{t2}t_{i2}+\beta_{t3}t_{i3}+\sum_{k=1}^{K}\beta_{k}X_{ikt}+\alpha_{i}+\theta_{m_{t}}^{\left(3\right)}+\sum_{j\epsilon \;Ins(i_{t})}w_{i_{t}j}^{\left(4\right)}\gamma_{j}^{\left(4\right)}$$ (3)

where *t* (*t* = 1,2,3) denotes semester, $logit\;(P(y_{i_{t}|i,\;Ins(i_{t}),maj(m_{t})}^{*}\geq 3))$ is the log odds of the probability of academic success at semester *t* given the random effects of student *i* (*i* = 1,…,*N*), instructors *j* in *Ins*(*i*_*t*_), and major *m*_*t*_. The coefficient *β*_0_ represents the intercept, *t*_*i*2_ and *t*_*i*3_ are dummy variables that take value 1 if data was collected at semesters 2 and 3, respectively, with *β*_*t*2_ and *β*_*t*3_ as their corresponding coefficients. The fixed effects are represented by *X*_*i*1*t*_,…,*X*_*iKt*_ in the model and *β*_1_,…,*β*_*K*_ are their regression coefficients. The random effect of student *i* taking classes at semester *t* is *α*_*i*_ and is distributed as $\alpha_{i}∼N(0,\sigma_{\alpha }^{2})$. Major pursued by student *i* at semester *t* is represented by *m*_*t*_, and $\theta_{m_{t}}^{\left(3\right)}$ represents its random effect which is distributed as $\theta_{m_{t}}^{\left(3\right)}∼N(0,\sigma_{\theta }^{2})$. The set *Ins*(*i*_*t*_) contains all the instructors that student *i* took classes with at semester *t*, $w_{i_{t}j}^{\left(4\right)}$ is the weight corresponding to instructor *j* on student *i* at semester *t*, $\gamma_{j}^{\left(4\right)}$ is the random effect associated with instructor *j*, such that $\gamma_{j}^{\left(4\right)}∼N(0,\sigma_{\gamma }^{2})$. In this model, it is assumed that the random effects for students, instructors and majors are independent and the error term for $y_{i_{t}|i,Ins(i_{t}),maj(m_{t})}^{*},\;\varepsilon_{i_{t}|i,Ins(i_{t}),maj(m_{t})}^{*}∼logistic\left(0,\frac{\pi^{2}}{3}\right)$. Thus, the probability of getting a B or better at semester *t*, for student *i*, taking classes with instructors in the set *Ins*(*i*_*t*_)⊂(1,…,*J*) and pursuing major *m*_*t*_ is

$$P(y_{i_{t}|i,Ins(i_{t}),maj(m_{t})}\geq 3)=\frac{e^{\beta_{0}+\beta_{t2}t_{2}+\beta_{t3}t_{3}+\sum_{k=1}^{K}\beta_{k}X_{ikt}+\alpha_{i}+\theta_{m_{t}}^{\left(3\right)}+\sum_{j\epsilon \;Ins(i_{t})}w_{i_{t}j}^{\left(4\right)}\gamma_{j}^{\left(4\right)}}}{1+e^{\beta_{0}+\beta_{t2}t_{2}+\beta_{t3}t_{3}+\sum_{k=1}^{K}\beta_{k}X_{ikt}+\alpha_{i}+\theta_{m_{t}}^{\left(3\right)}+\sum_{j\epsilon \;Ins(i_{t})}w_{i_{t}j}^{\left(4\right)}\gamma_{j}^{\left(4\right)}}}$$ (4)

Then, the total response variance for student *i* at semester *t* [25]

$$var{(y_{i_{t}|i,Ins(i_{t}),maj(m_{t})})=\sigma_{\alpha }^{2}+\sigma_{\theta }^{2}+\sigma }_{\gamma }^{2}\sum_{j\epsilon \;Ins(i_{t})}({w_{i_{t}j}^{\left(4\right)})}^{2}+\frac{\pi^{2}}{3}$$ (5)

We compute the variance partition coefficient (VPC) for majors as

$$VPC_{maj}=\frac{\sigma_{\theta }^{2}}{\sigma_{\alpha }^{2}+\sigma_{\theta }^{2}+\sigma_{\gamma }^{2}\;\sum_{j\epsilon \;Ins(i_{t})}({w_{i_{t}j}^{\left(4\right)})}^{2}+\frac{\pi^{2}}{3}}$$

and for instructors as

$$VPC_{ins}=\frac{\sigma_{\gamma }^{2}\;\sum_{j\epsilon \;Ins(i_{t})}({w_{i_{t}j}^{\left(4\right)})}^{2}}{\sigma_{\alpha }^{2}+\sigma_{\theta }^{2}+\sigma_{\gamma }^{2}\;\sum_{j\epsilon \;Ins(i_{t})}({w_{i_{t}j}^{\left(4\right)})}^{2}+\frac{\pi^{2}}{3}}$$

to estimate the proportion of the response variance attributed to majors and instructors respectively.

As mentioned earlier, when modeling academic success for student *i* at semester *t*, we only accounted for the effects of instructors with whom the student took classes and the major the student was enrolled in at that semester. Thus, at each semester, students were multiple members of the instructors’ classification and were completely nested within the major classification. We achieved this through the weights in the model. At each semester *t*, students had a set of instructors, *Ins*(*i*_*t*_). In the multiple membership multiple classification model, the weights for the instructors in the set *Ins*(*i*_*t*_) were calculated as the ratio of one divided by the number of instructors the student had classes with at that semester (the number of elements in *Ins*(*i*_*t*_)), such that $w_{i_{t}j}^{\left(4\right)}=\frac{1}{\#of\;instructors\;in\;set\;Ins(i_{t})}$. Thus, for instructors that did not teach the student at semester *t* the weights were 0, even if they taught students at previous or later semesters. In situations where an instructor taught a student again, the weight for that instructor was recalculated based on the number of instructors at that new semester. Similarly, for majors, we only accounted for the effects of the major pursued at semester *t*. If a student changed majors from one semester to another then we just changed the major in the model for the next semester. Our model assumed that major was the same at all times during a semester, but it did not assume that major did not change throughout the data collection period. However, we did not consider major to be a multiple membership classification in our data, since we assumed that the major pursued at a previous or later semester did not affect GPA at semester *t*. Going back to Fig 2, when modeling *P*(*GPA*_1_≥3), the weights for *Instructor*_1*s*_ and *Instructor*_1*k*_ were $\frac{1}{2}$ each, while for all other instructors including *Instructor*_2*j*_ and *Instructor*_2*l*_ the weights were zero. A similar approach was followed when modeling *P*(*GPA*_2_≥3). In other words, for each GPA we only had non-zero weights for instructors and majors that were connected to that GPA through a dotted line.

We used Bayes estimates to fit the model and to obtain posterior distributions for the regression coefficients, and variances corresponding random effects for students, instructors and majors.

### Bayes estimates

We present a MMMC logistic regression model that accounts for the effects of majors through a prior distribution and the effects of instructors with weights. We used the MCMC method of estimation and the Metropolis-Hastings sampling using adaptive method in MLwiN 3.0 software, to obtain our results [26]. We used the inverted gamma (2.5, 4.5) prior distribution for the variances of the random effects for students, $\sigma_{\alpha }^{2}$, majors, $\sigma_{\gamma }^{2}$, and instructors, $\sigma_{\theta }^{2}$. As the inverted gamma distribution has support on positive values, it guarantees that we only draw positive values for the variances of the random effects. However, we used normal priors for the regression coefficients. The intercept *β*_0_, and the regression coefficients *β*_*t*2_ and *β*_*t*3_, for semesters’ dummy variables *t*_2_ and *t*_3_ had non-informative priors, N(0,10000).

Also, we had information that international students [*N*(0.5,1)] obtained better GPAs than in-state residents and that out of state students [*N*(−0.5,1)] had lower GPAs than in-state residents. Freshmen, sophomores and juniors had lower GPAs than seniors [*N*(−0.5,1)]. The number of classes [*N*(0.5,1)] college students took during their first year increased their GPA [27]. Student athletes [*N*(−0.5,1)] had lower GPAs than non-students athlete [28]. Students with disabilities [*N*(−0.5,1)] obtained lower semester GPAs than students without disabilities [29]. These priors with a binomial likelihood provided a posterior from which the regression coefficients and the variance estimates were obtained. This was conducted with a Markov Chain Monte Carlo sampling algorithm. The initial values for the Markov Chain were obtained by fitting a multilevel model that only included the first instructor and ignored the multiple membership structure of the data. Then, the initial estimates were calculated through an Iterative Generalized Least Squares (IGLS) procedure.

We used 2,000 burn in samples for modeling GPA 3.0 or better [*Model3*.*0*], followed by 320,000 iterations with a thinning of 8, resulting in a chain length of 40,000. While for modeling of GPA 2.0 or better [*Model2*.*0*], we used a burn in of 2,000 followed by 800,000 iterations and thinning of 10, resulting in a chain length of 80,000. The effective sample size (ESS) for all parameter estimates was larger than 100 [30].

We explored the fit of these two models, [*Model3*.*0*] and [*Model2*.*0*]. Each model had three random effects: students, instructors and majors. In addition, we fitted models with and without those random effects, resulting in information for eight models. Thus, we compared the fit of eight models. The best model was determined based on the lowest Bayesian Deviance Information Criterion (DIC). This Bayesian fit index is defined as $DIC=\bar{D}+p_{D}$; the sum of the Posterior Mean Deviance $\left(\bar{D}\right)$, a Bayesian measure of fit or adequacy, and the effective number of parameters in the model *p*_*D*_, which is a measure of model complexity [31]. A difference in values between DIC for two models greater than seven units provides strong evidence in favor of the model with smaller DIC [23]. Comparisons of DIC values for the eight models with different combinations of the three random effects can be performed using Tables A1 and A2 in S1 Appendix corresponding to [*Model3*.*0*] and [*Model2*.*0*], respectively. For both measures of success, the DIC supported as the model of choice the Bayesian model with random effects for students, instructors and majors.

**Fig 3:**
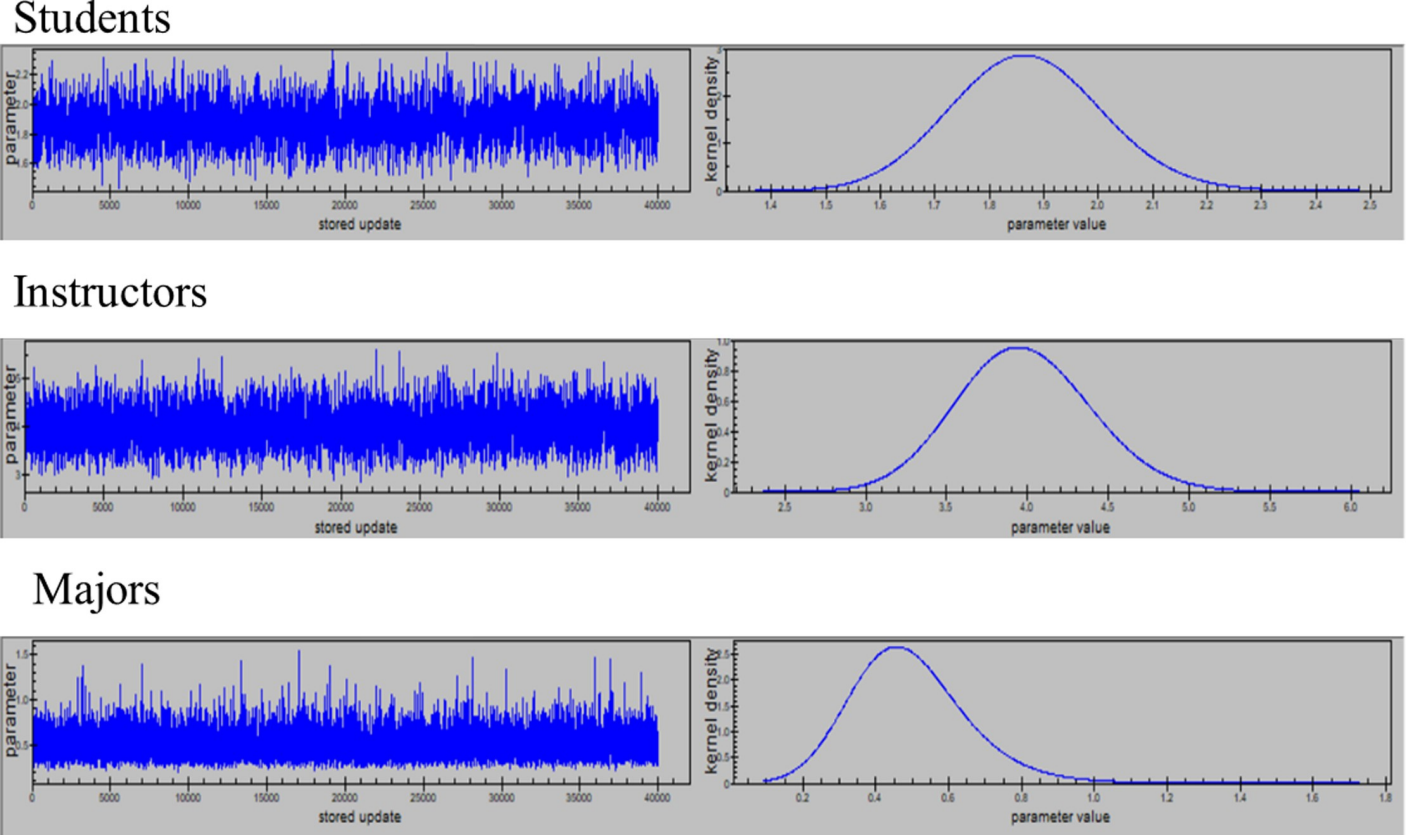
Markov Chains and posterior distributions for variance components when modeling probability of getting GPA 3.0 or better.

**Fig 4:**
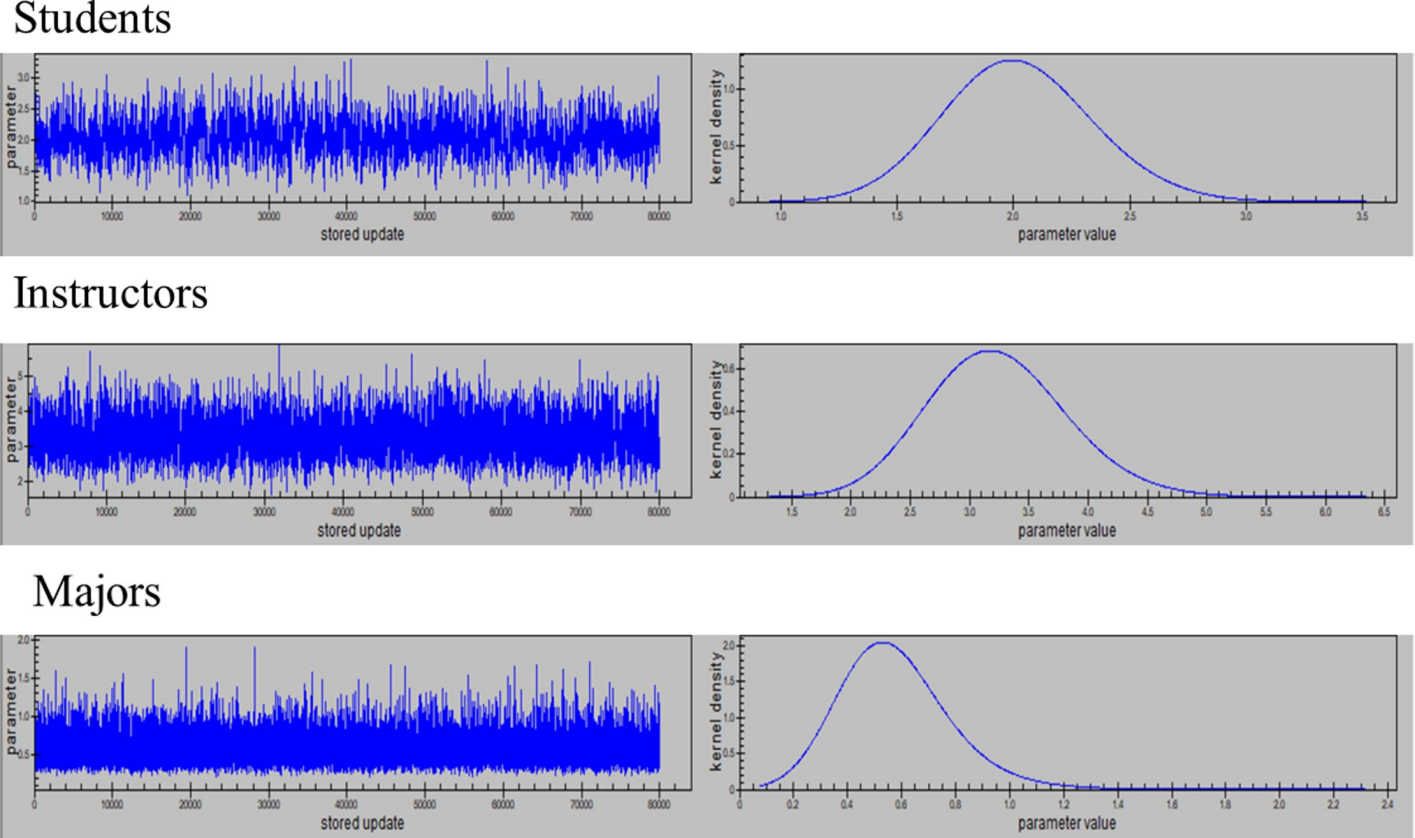
Markov Chains and posterior distributions for variance components when modeling probability of getting GPA 2.0 or better.

Each model provided evidence of a stationary Markov Chain after the burn in period and convergence to the same posterior region. The Bayes estimates were obtained using the squared error loss, thereby, providing posterior means. Markov chains for variance of random effects (students, instructors and majors), regression coefficients and their posterior distributions are presented, Figs 3 and 4 and Figs A1 and A2 in S1 Appendix.

The Metropolis Hastings sampling algorithm that gives rise to these estimates is summarized [11]:

Let ***y*** = (***y***_**1**_,…,***y***_***n***_) denote the vector of outcomes, ***β*** is the vector of regression coefficients for the fixed effects, ***α*** is the vector of random effects for students, ***θ***^(3)^ is the vector of random effects for majors, ***γ***^(4)^ is the vector of random effects for instructors. The posterior distribution from which the draws are taken, is:

$$p(\beta ,\alpha ,\theta^{\left(3\right)},\gamma^{\left(4\right)},\sigma_{\alpha }^{2},\sigma_{\theta }^{2},\sigma_{\gamma }^{2}|y)\propto p(y|\beta ,\alpha ,\theta^{\left(3\right)},\gamma^{\left(4\right)},\sigma_{\alpha }^{2},\sigma_{\theta }^{2},\sigma_{\gamma }^{2})\;p(\alpha |\sigma_{\alpha }^{2})\;p(\theta^{\left(3\right)}|\sigma_{\theta }^{2})\;p(\gamma^{\left(4\right)}|\sigma_{\gamma }^{2})\;p(\beta )\;p(\sigma_{\alpha }^{2})\;p(\sigma_{\theta }^{2})\;p(\sigma_{\gamma }^{2})$$

Let ${logit(\pi }_{i_{t}})$ be the systematic component with link function logit, such that

$${logit\;(\pi }_{i_{t}})=\beta_{0}+\beta_{t2}t_{i2}+\beta_{t3}t_{i3}+\sum_{k=1}^{K}\beta_{k}X_{ikt}+\alpha_{i}^{\left(2\right)}+\theta_{m_{t}}^{\left(3\right)}+\sum_{j\epsilon \;Ins(i_{t})}w_{i_{t}j}^{\left(4\right)}\gamma_{j}^{\left(4\right)}$$

Thus, at each iteration *s*, we had the subsequent steps:

1. Update ***β*** using univariate random walk metropolis as follows: for *l* = *t*_2_,*t*_3_,1,…,*k* and with ***β***_(−*l*)_ representing ***β*** without component *l*
  $\beta_{l}(s)=\beta_{l}^{*}$ with probability $\min\;\left(1,\frac{p(\beta_{l}^{*}|y,\;\alpha ,\theta^{\left(3\right)},\;\gamma^{\left(4\right)},\;\beta_{\left(-l\right)})}{p(\beta_{l}(s-1)|y,\;\alpha ,\;\theta^{\left(3\right)},\;\gamma^{\left(4\right)},\beta_{\left(-l\right)})}\right)$
  = *β*_*l*_(*s*−1) otherwise,
  where $\beta_{l}^{*}∼N(\beta_{l}(s-1),\sigma_{l}^{2})$ and
  $$p(\beta_{l}|y,\alpha ,\theta^{\left(3\right)},\gamma^{\left(4\right)},\beta_{\left(-l\right)})\propto \prod_{i_{t}}{\left(1+e^{-\pi_{i_{t}}}\right)}^{-y_{i_{t}}}\;{\left(1+e^{\pi_{i_{t}}}\right)}^{y_{i_{t}}-1}$$
2. Update students’ random effects, *α*_*i*_, using univariate random walk metropolis for *i* = 1,…,*N*;
  $\alpha_{i}(s)=\alpha_{i}^{*}$ with probability $min\;\left(1,\frac{p(\alpha_{i}^{*}|y,\;\theta^{\left(3\right)},\;\gamma^{\left(4\right)},\;\beta ,\;\sigma_{\alpha }^{2})}{p(\alpha_{i}(s-1)|y,\;\theta^{\left(3\right)},\;\gamma^{\left(4\right)},\;\beta ,\;\sigma_{\alpha }^{2})}\right)$
  = *α*_*i*_(*s*−1) otherwise
  where $\alpha_{i}^{*}∼N(\alpha_{i}(s-1),\sigma_{\alpha }^{2})$ and
  $$p(\alpha_{i}|y,\;\theta^{\left(3\right)},\;\gamma^{\left(4\right)},\beta ,\;\sigma_{\alpha }^{2})\propto exp\left\{-\frac{1}{2\sigma_{\alpha }^{2}}\alpha_{i}^{2}\right\}\prod_{i_{t},student=i}{\left(1+e^{-\pi_{i_{t}}}\right)}^{-y_{i_{t}}}{\left(1+e^{\pi_{i_{t}}}\right)}^{y_{i_{t}}-1}$$
3. Update the majors’ random effects, $\theta_{m}^{\left(3\right)}$, using univariate random walk metropolis for *m* = 1,…,*M*;
  $\theta_{m}^{\left(3\right)}(s)=\theta_{m}^{(3)*}$ with probability $min\;\left(1,\frac{p(\theta_{m}^{(3)*}|y,\;\alpha ,\;\gamma^{\left(4\right)},\;\beta ,\;\sigma_{\theta }^{2})}{p(\theta_{m}^{\left(3\right)}(s-1)|y,\;\alpha ,\;\gamma^{\left(4\right)},\beta ,\;\sigma_{\theta }^{2})}\right)$
  $=\theta_{m}^{\left(3\right)}(s-1)$ otherwise, where $\theta_{m}^{(3)*}∼N(\theta_{m}^{\left(3\right)}(s-1),\sigma_{\theta }^{2})$ and
  $$p(\theta_{m}^{\left(3\right)}|y,\;\alpha ,\;\gamma^{\left(4\right)},\beta ,\;\sigma_{\theta }^{2})\propto exp\left\{-\frac{1}{2\sigma_{\theta }^{2}}{\left(\theta_{m}^{(3)}\right)}^{2}\right\}\prod_{i_{t},major=m}{\left(1+e^{-\pi_{i_{t}}}\right)}^{-y_{i_{t}}}{\left(1+e^{\pi_{i_{t}}}\right)}^{y_{i_{t}}-1}$$
4. Update the instructors’ effects, $\gamma_{j}^{\left(4\right)}$, using univariate random walk metropolis for *j* = 1,…,*J*
  $\gamma_{j}^{\left(4\right)}(s)=\gamma_{j}^{(4)*}$ if with probability min $\left(1,\frac{p(\gamma_{j}^{(4)*}|y,\;\alpha ,\;\theta^{\left(3\right)},\beta ,\;\sigma_{\gamma }^{2})}{p(\gamma_{j}^{\left(4\right)}(s-1)|y,\;\alpha ,\;\theta^{\left(3\right)},\;\beta ,\;\sigma_{\gamma }^{2})}\right)$
  $=\gamma_{j}^{\left(4\right)}(s-1)$ otherwise
  where $\gamma_{j}^{(4)*}∼N(\gamma_{j}^{\left(4\right)}(s-1),\sigma_{\gamma }^{2})$ and
  $$p(\gamma_{j}^{\left(4\right)}|y,\alpha ,\theta^{\left(3\right)},\beta ,\sigma_{\gamma }^{2})\propto exp\left\{-\frac{1}{2\sigma_{\gamma }^{2}}{\left(\gamma_{j}^{\left(4\right)}\right)}^{2}\right\}\prod_{i_{t},j\epsilon Ins(i_{t})}{\left(1+e^{-\pi_{i_{t}}}\right)}^{-y_{i_{t}}}{\left(1+e^{\pi_{i_{t}}}\right)}^{y_{i_{t}}-1}$$
5. Update students’ effects’ variance, $\sigma_{\alpha }^{2}$, by drawing from the gamma full conditional distribution of
  $$\sigma_{\alpha }^{2}:p(\sigma_{\alpha }^{2}|y,\beta ,\alpha ,\theta^{\left(3\right)},\gamma^{\left(4\right)},\sigma_{\theta }^{2},\sigma_{\gamma }^{2})∼Inv-Gamma\left(\frac{N+\nu_{i}}{2},\frac{1}{2}(\sum_{i=1}^{N}\alpha_{i}^{2}+\nu_{i}s_{i}^{2})\right)$$
6. Update majors’ effects’ variance, $\sigma_{\theta }^{2}$, by drawing from the gamma full conditional distribution of
  $$\sigma_{\theta }^{2}:p(\sigma_{\theta }^{2}|y,\beta ,\alpha ,\theta^{\left(3\right)},\gamma^{\left(4\right)},\sigma_{\alpha }^{2},\sigma_{\gamma }^{2})∼Inv-Gamma\left(\frac{M+\nu_{m}}{2},\frac{1}{2}(\sum_{m=1}^{M}{\left(\theta_{m}^{\left(3\right)}\right)}^{2}+\nu_{m}s_{m}^{2})\right)$$
7. Update instructors’ effects’ variance, $\sigma_{\gamma }^{2}$, by drawing from the gamma full conditional distribution of
  $$\sigma_{\gamma }^{2}:p(\sigma_{\gamma }^{2}|y,\beta ,\alpha ,\theta^{\left(3\right)},\gamma^{\left(4\right)},\sigma_{\alpha }^{2},\sigma_{\theta }^{2})∼Inv-Gamma\left(\frac{J+\nu_{j}}{2},\frac{1}{2}(\sum_{j=1}^{J}{\left(\gamma_{j}^{\left(4\right)}\right)}^{2}+\nu_{j}s_{j}^{2})\right)$$

### Ethics and approval

The Institutional Review Board determined that the protocol is considered exempt pursuant to Federal Regulations 45CFR46 (1) Educational settings on 4/23/2019. Data were fully anonymized before we accessed them and informed consent was waivered by the IRB.

## Results

We fitted MMMC logistic regression models to study student success, *Model2*.*0* (GPA of 2.0 or better) and *Model3*.*0* (GPA of 3.0 or better), with Bayesian estimates. The advantage of Bayes’ estimates was realized as there was the added complexity of sparsity because of the low number of student athletes and students using Disability Resource Services (DRS) in the dataset. Frequentist models were attempted to fit to the data, but by virtue of their likelihoods, suffered from non-convergence issues. We used data for three consecutive semesters consisting of 24,551 semester GPAs for 14,103 undergraduate students enrolled in a college at a large state university from May 2014 to May 2015. Semesters were included in the model as fixed effects to allow for more courses and to address any varying rates of academic success across semesters. The covariates of interest included: student residency classification, student academic level, student-athlete or not, use of Disability Resources or not, and the number of classes enrolled in the semester.

Approximately 59% of students were in-state residents, 37% of students were out-of-state residents and 4% of students were international students. Approximately 1% of the students in the data were athletes. Less than 3% of students made use of DRS. Each semester had approximately 67% of students with GPA B or better, and 94% of students with GPA 2.0 or better, Table 1.

**Table 1:** Percentage success over semester.

Semester	Model3.0 = % 3.0 or better	Model2.0 = % 2.0 or better
Semester 1	69%	95%
Semester 2	67%	94%
Semester 3	66%	93%

There were 40 majors, 1,867 courses and 2,802 instructors. Approximately 3% of instructors were teaching assistants. The percentages of GPAs greater than or equal to 2.0 and 3.0 were similar in all semesters. Approximately 50% of the students were enrolled in business majors, with the remaining student majors as follows: 10% in accountancy-related majors or finance-related majors, approximately 7% in marketing-related majors, 7% in management programs, approximately 6% in supply chain management, 3% in computer information systems, 2% in economics, and less than 1% in agribusiness. Descriptive statistics for variables in model per semester are provided in Table 2.

**Table 2:** Descriptive statistics for data.

Variable	Semester 1	Semester 2	Semester 3
Residency Status (%)			
In-state	65.62	61.54	56.97
Out of state	27.42	33.94	40.73
International	6.95	4.52	2.3
Academic level (%)			
Freshman	2.78	20.52	20.99
Sophomore	23.02	18.58	19.8
Junior	32.4	25.07	25.67
Senior	41.8	35.82	33.53
Average number of classes (SD)	4.65 (1.33)	4.70 (1.32)	4.76 (1.33)
Athlete (%)	0.88	1.05	0.82
DRS (%)	2.53	2.57	2.35

It was rare to find a student who took more than one class from the same instructor in a semester. Thus, we assumed that each of the instructors contributed equally to a student’s semester GPA, regardless of the number of classes taken. Therefore, if student *i* had *n* instructors at semester *t*, then the weight $w_{i_{t}j}$ for student *i* and instructor *j* was 1nfor each instructor. So, the weights were assigned equally to the instructors. For students who only had one instructor, the corresponding weight was set to one. For students who had more than one semester of data, at semester *t*, non-zero weights were given only to instructors who taught the students at that semester. Weights for all other instructors including those that taught students at previous or later semesters where equal to zero.

We obtained posterior odds ratios with 95% credible intervals, posterior regression coefficients, variance components, and the effective sample sizes for modeling the probability of success, Table 3. Reported estimates for regression coefficients and variance components correspond to the means of their posterior distributions. Most covariates impacted both probabilities of success (GPA of 3.0 or greater and GPA of 2.0 or greater) in the same way, except for residency status and use of DRS. Variations between out-of-state and in-state students were not significant in measuring success at 3.0 while use of DRS was significant in measuring success at 3.0. We also obtained the VPCs for instructors and majors for situations with different number of instructors, Table 4.

**Table 3:** Modeling GPA 3.0 or better/ GPA 2.0 or better.

Parameter	GPA 3.0 or better			GPA 2.0 or better		
	Estimate (SE)	OR (95% CI)	ESS	Estimate (SE)	OR (95% CI)	ESS
**Intercept**	1.793 (0.159)		718	4.634 (0.234)		1653
**Semester**	** **			** **		
Fall 2014	0.095 (0.058)	1.100 (0.981, 1.234)	10517	0.208 (0.107)	1.231 (0.998, 1.519)	24787
Spring 2015	-0.171 (0.067)	0.843 (0.739, 0.962)	7643	-0.416 (0.113)	0.660 (0.528, 0.821)	15474
**Residency Status**	** **		** **	** **		** **
Out of State	-0.051 (0.049)	0.950 (0.862, 1.047)	16961	0.276 (0.083)	1.318 (1.121, 1.550)	44964
International	-0.495 (0.117)	0.610 (0.486, 0.769)	21137	-0.116 (0.220)	0.890 (0.582, 1.381)	60481
**Academic level**	** **		** **	** **		** **
Freshmen	-1.350 (0.096)	0.259 (0.215, 0.312)	6945	-1.850 (0.148)	0.157 (0.117, 0.210)	11852
Sophomore	-0.496 (0.079)	0.609(0.522, 0.711)	7402	-0.924 (0.134)	0.397 (0.305, 0.516)	15897
Junior	-0.210 (0.058)	0.811 (0.723, 0.907)	11467	-0.352 (0.110)	0.703 (0.567, 0.875)	24525
**Class count**	0.437 (0.018)	1.548 (1.495, 1.603)	19543	0.627 (0.031)	1.872 (1.765, 1.990)	8188
**Athlete**	0.386 (0.225)	1.471 (0.951, 2.286)	28567	1.340 (0.485)	3.819 (1.550, 10.444)	71143
**DRS**	-0.297 (0.135)	0.743 (0.571, 0.969)	25912	0.132 (0.225)	1.141 (0.740, 1.790)	67414
**Variance**	**Estimate (SE)**	**95% CI**	**ESS**	**Estimate (SE)**	**95% CI**	**ESS**
Students	1.869 (0.124)	(1.634, 2.122)	2071	2.022 (0.289)	(1.456, 2.587)	1105
Instructors	3.979 (0.361)	(3.312, 4.727)	4863	3.239 (0.518)	(2.224, 4.254)	3491
Majors	0.493 (0.134)	(0.293, 0.809)	28488	0.574 (0.169)	(0.244, 0.905)	57789

CI: Credible Interval

**Table 4:** Variance partition coefficients for GPA 3.0 or better/ GPA 2.0 or better.

# of instructors	variance component	GPA 3.0 or greater (%)	GPA 2.0 or greater (%)
1 instructor	Major	5.12	6.29
	Instructor	41.31	35.49
2 instructors	Major	6.45	7.65
	Instructors	26.03	21.58
3 instructors	Major	7.06	8.24
	Instructors	19.01	15.50
4 instructors	Major	7.42	8.57
	Instructors	14.96	12.09
5 instructors	Major	7.65	8.78
	Instructors	12.34	9.91
6 instructors	Major	7.81	8.93
	Instructors	10.50	8.40
7 instructors	Major	7.92	9.04
	Instructors	9.14	7.29
8 instructors	Major	8.02	9.12
	Instructors	8.09	6.43

Using *Model3*.*0*, the 95% credible interval for odds ratio for class count was (1.495, 1.603). Thus, increasing number of classes taken during a semester had a significant positive impact on the probability of academic success. Similar results were obtained with *Model2*.*0* and 95% credible interval for class count was (1.765, 1.990). In addition, for *Model2*.*0* out of state residency had 95% credible intervals for odds ratios as (1.121, 1.550). Thus, suggesting that out-of-state students had a positive significant impact on academic success as opposed to in-state students.

We also obtained 95% credible intervals for international students (0.486, 0.769), freshmen (0.215, 0.312), sophomores (0.522, 0.711), and juniors (0.723, 0.907), and students using DRS (0.571, 0.969) under *Model3*.*0*. The seniors and those not using DRS were more likely to have academic success based on the *Model3*.*0*. International students were significantly less likely to have academic success than in-state residents. Freshmen, sophomores and juniors were significantly less likely to have academic success when compared to seniors. Students utilizing DRS were significantly less likely to achieve academic success compared to those who did not use the services. Similar results were obtained based on a fit of *Model2*.*0*. Freshmen (0.117, 0.210), sophomore (0.305, 0.516), and junior (0.567, 0.875) students were significantly less likely to have academic success as compared to seniors. Athletes (1.550, 10.444) were significantly more likely to achieve a 2.0 GPA than non-athletes.

The 95% credible intervals for the odds ratios showing no significance included: international (0.582, 1.381), and DRS (0.740, 1.790) based on *Model2*.*0*. Thus, there was no significant difference in the probability of success between international and in-state students. We found no difference in academic success rate between DRS and non-DRS students with *Model2*.*0*. Similarly, for *Model3*.*0* covariates with 95% credible intervals for odds ratios with no significant results were as follows: out-of-state (0.862, 1.047) and athlete (0.951, 2.286).

According to *Model3*.*0*, the posterior variance for instructors (random effects) was significant in determining academic success attributable to the instructor, with a standardized value of 11.022. Similarly, the posterior variance for majors (random effects) was significant and indicated that a substantial portion of academic success is attributable to the major; it had a standardized value of 3.679. The VPCs suggested that instructors contributed overwhelmingly to overall success. Similar results were obtained with the *Model2*.*0*. That model showed the posterior variance for instructors and majors had standardized values of 6.253 and 3.396 respectively.

## Discussion

Several studies have shown that international students struggle academically on account of the different cultural and sometimes language barriers that exist [32–34]. We found that out-of-state and international students were less likely to get a GPA of 3.0 or better as compared to in-state students. However, being an out-of-state student had a significant impact on the probability of getting GPA of 2.0 or higher. International students were less likely to get a GPA of 3.0 or better and also less likely to get a GPA of 2.0 or better than in-state students. Being an international student had a significant impact on the probability of getting a GPA of 3.0 or better, but it did not make a significant difference on the probability of getting a GPA of 2.0 or better.

Freshmen, sophomores and juniors were less likely to be successful as compared to seniors. The effects were stronger when modeling a GPA of 2.0 or better than when modeling GPA of 3.0 or better. Thus, if a student reaches the senior year, then we can expect the student to do better as he or she proceeds to graduation. Also, we found that those who took more classes performed better academically than those who did not. However, this may be due to the fact that those who take more classes are also those who perform well.

Athletes, a relatively small group of students, did not have a disadvantage regarding the probability of success. In particular, there was no significant difference in academic success as measured by getting a 3.0 or better GPA between student athletes and non-athlete students, and athlete students were significantly more likely to get a GPA of 2.0 or better than non-athletes. However, this may be obscured, as the ratio of the population of athletes to the population of non-student athletes is very small. This finding contradicts the belief held by most academic administrators and the general population that student athletes’ academic performances are not as good as that of non-student athletes. It also suggests that the programs and the other mandates put in place by the university and institutions overseeing the performance of athletes are making a difference. On the other hand, more is needed to be done concerning disability resources. We found that students who use disability resources are less likely to be academically successful as compared to those students who do not. Academic officials should continue to fund programs that improve conditions for students with disabilities and help them succeed.

We found that there was high variability in success rates between majors and instructors. Academic success depends on the classes taken and the instructors that delivered the material. Students are more likely to be successful based on the major and the person who teaches in these majors.

## Conclusion

We investigated the impact of several student characteristics that are not normally used when modeling college student academic success, such as out-of-state, in-state or international residency status, simultaneously. While several researchers have investigated how international students perform academically, their studies are based on certain subpopulations rather than altogether. This approach is commonly used in some analyses focused on first-year college students, but not looking at all classes simultaneously.

Often researchers ignore the multilevel structure of the design, which may consist of different levels of correlation (e.g. students, majors and instructors). Hence, they analyze the levels of correlation separately. Such an approach negates any conclusions regarding instructors or majors across the broad spectrum. We use such level of information (e.g., majors and instructors) as random effects. This approach allows one to report on the impact of majors and the impact of instructors on students’ success.

Studies that report on the impact of instructors on college students’ academic success have usually done so by classifying instructors into one of several categories and treating them as fixed effects. However, this approach limits the ability to measure their impact. In using instructors and majors as random effects, one accounts for all unmeasurable effects. The fact that we do not conduct separate analyses for each major or each instructor but treat these as random effects renders our results generalizable.

Several studies have investigated the influence of socioeconomic factors (i.e. race, first-generation status, and whether students work part-time or full-time while attending college, etc.) or how academic success is impacted by economics in other ways (e.g., free academic resources in classes vs. students required to buy books, etc.). Such studies usually do not account for the unmeasurable differences that occur due to majors or instructors because the authors conduct separate analyses for each major or instructor. This research represents an alternative approach to modeling academic success in college environments. These models give us a unique opportunity of identifying the effects of student characteristics and immeasurable factors (instructors and majors) on academic success. In so doing, we include the multilevel non-hierarchical structure of the data that leads to correlation among observations at different levels.

Improved graduation and retention rates are key components at public universities that compete for prospective students and state funding. Thus, improved student performance is of utmost importance for universities concerned with sustaining their competitive edge. As such, academic officials consistently looking to find new techniques that will place their institutions at or near the top of the competition. Thus, it is extremely important to identify factors affecting student performance.

## Supporting information

S1 Appendix Tables for DIC comparisons and posterior distributions of regression coefficients. The document includes tables with DIC values different combinations of random effects for both models and the Markov Chains and posterior distributions for the regression coefficients of both models.
(PDF)
