# Supplementary material for: Bayesian multiple membership multiple classification logistic regression model on student performance with random effects in university instructors and majors
Source: PLoS One. 2020 Jan 30;15(1):e0227343. doi: 10.1371/journal.pone.0227343 (PMC6992165; doi:10.1371/journal.pone.0227343)
Supplement: S1 Appendix — The document includes tables with DIC values different combinations of random effects for both models and the Markov Chains and posterior distributions for the regression coefficients of both models. (PDF) [file pone.0227343.s001.pdf]

S1 Appendix. Tables for DIC comparisons and posterior distributions of regression coefficients

Table A1. Model fit statistics for 3.0 or better GPA	
**Model**	**DIC**
No random effects	29124.208
Only majors as random effect	27535.775
Only instructors as random effects	27048.757
Only students as random effects	27513.903
Only majors and instructors as random effects	26846.280
Only majors and students as random effects	26936.783
Only students and instructors as random effects	25472.770
Only student, majors and instructors as random effects	25276.027

Table A2. Model fit statistics for 2.0 or better GPA	
**Model**	**DIC**
No random effects	9962.302
Only majors as random effect	9585.453
Only instructors as random effects	9487.459
Only students as random effects	9463.320
Only majors and instructors as random effects	9459.936
Only majors and students as random effects	9192.868
Only students and instructors as random effects	8907.255
Only student, majors and instructors as random effects	8881.786

FIGURE A1. Markov Chains and posterior distributions for covariates' coefficients when modeling probability of getting GPA 3.0 or better

Fall 2014

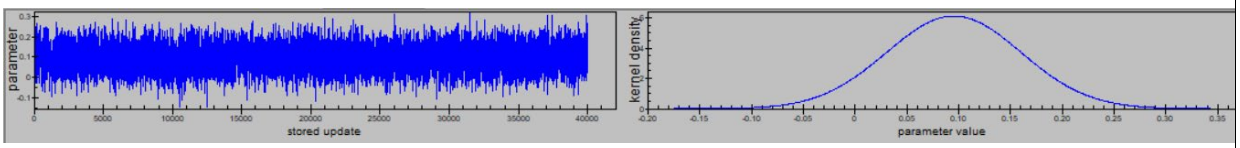

Spring 2015

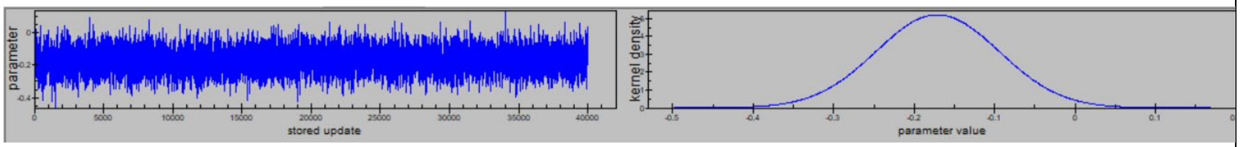

Out of state

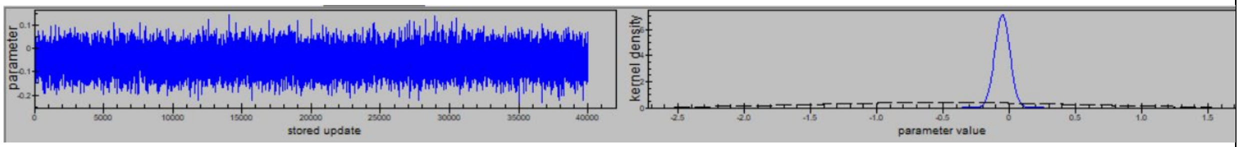

International

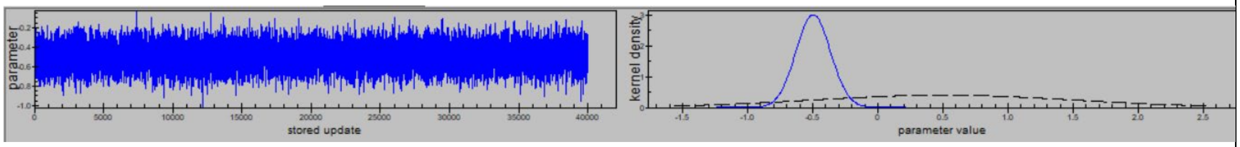

Freshman

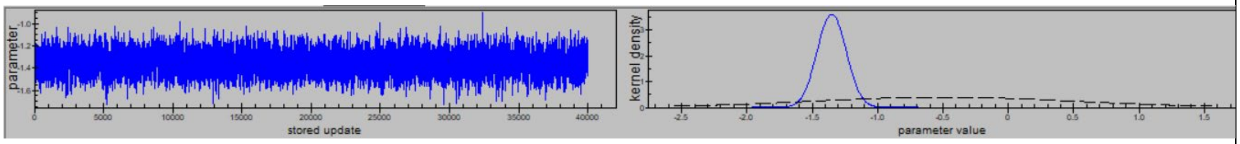

Sophomore

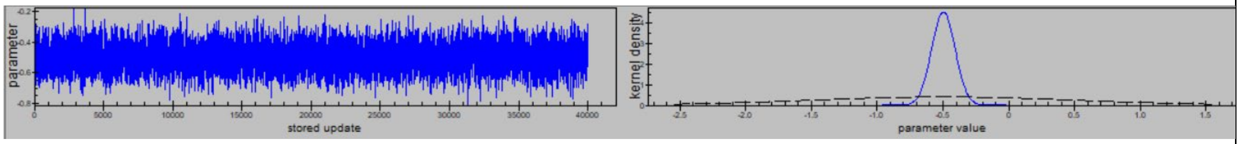

Junior

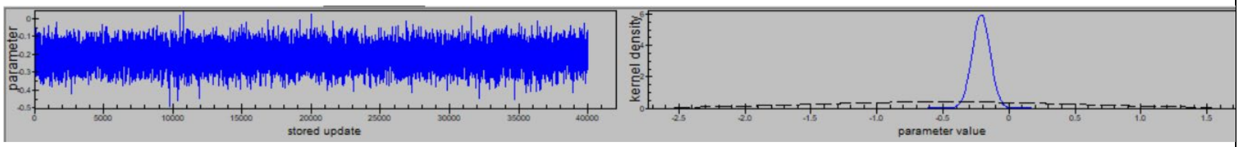

Class count

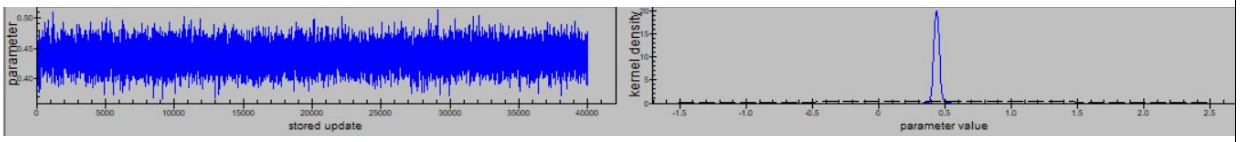

Athlete

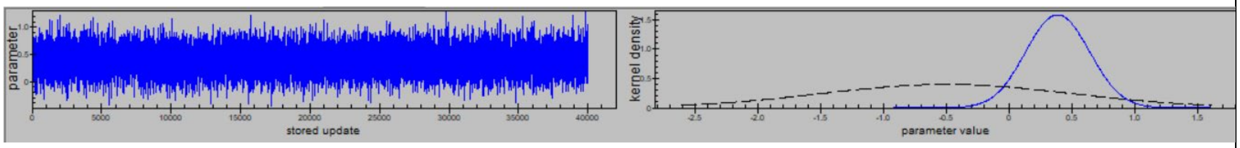

DRS

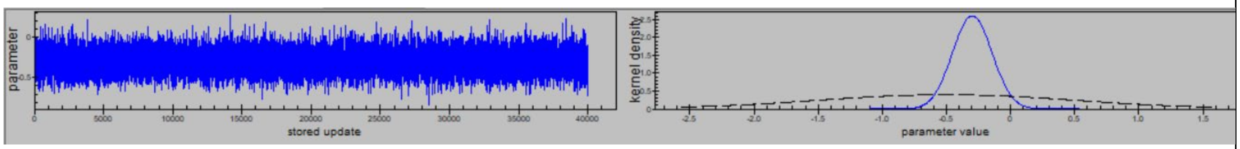

**Note: Blue curve represents posterior density and black curve represent prior distribution

Figure A2: Markov Chains and posterior distributions for covariates' coefficients when modeling probability of getting GPA 2.0 or better

Fall 2014

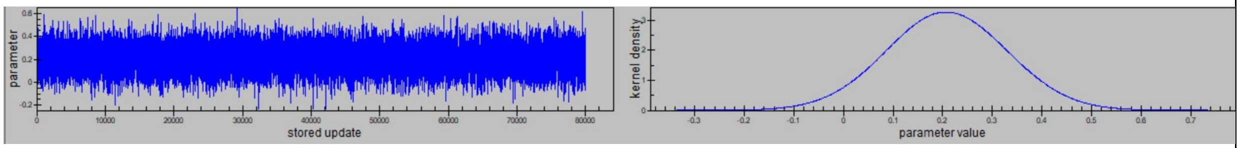

Spring 2015

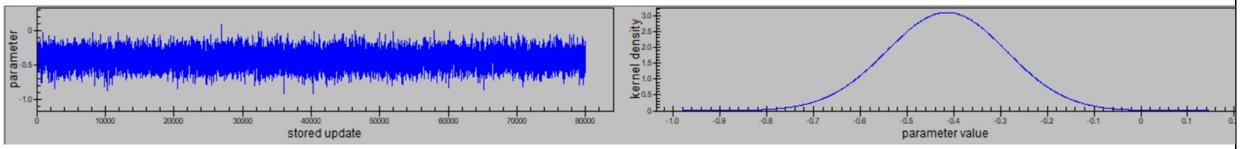

Out of state

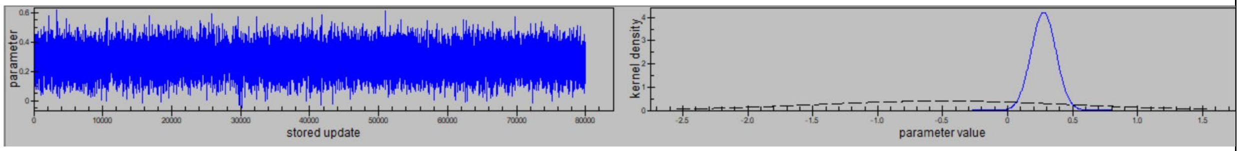

International

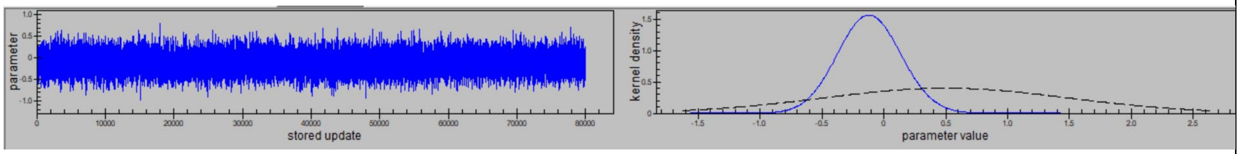

Freshman

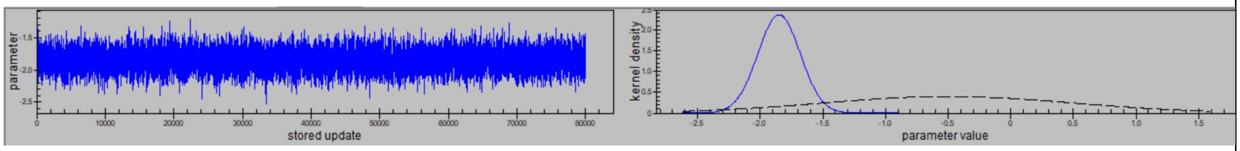

Sophomore

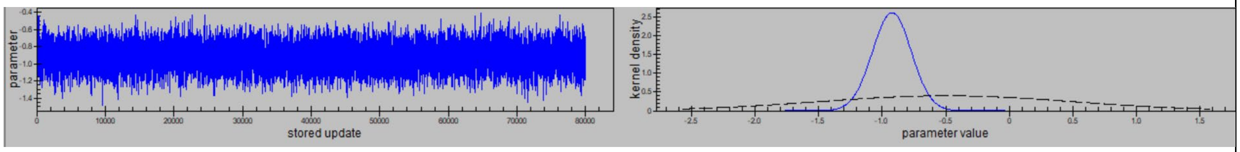

Junior

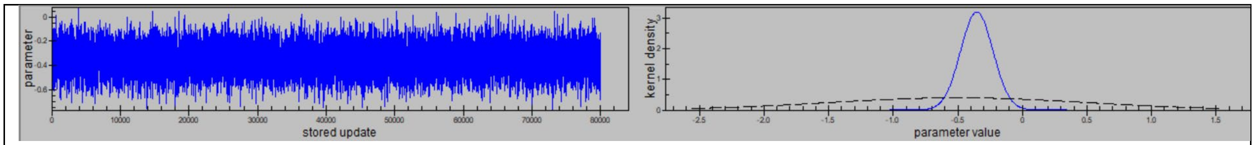

Class count

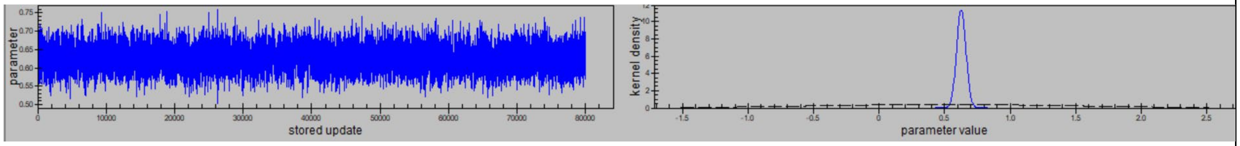

Athlete

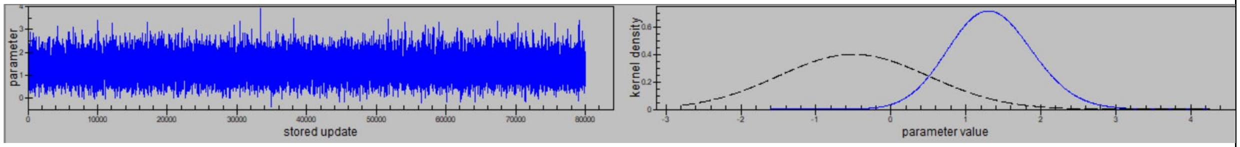

DRS

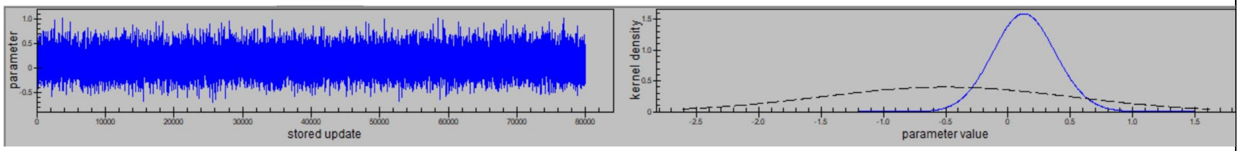

**Note: Blue curve represents posterior density and black curve represent prior distribution
